# The use of accelerometer-based wearable activity monitors in clinical settings: current practice, barriers, enablers, and future opportunities

**DOI:** 10.1186/s12913-021-07096-7

**Published:** 2021-10-08

**Authors:** Carol Maher, Kimberley Szeto, John Arnold

**Affiliations:** 1 Alliance for Research in Exercise, Nutrition and Activity (ARENA), Allied Health & Human Performance, University of South Australia, GPO Box 2471, SA 5001 Adelaide, Australia; 2 Innovation, Implementation and Clinical Translation in Health (IIMPACT in Health), Allied Health & Human Performance, University of South Australia, SA Adelaide, Australia

**Keywords:** Physical activity, Accelerometry, Exercise, Sedentary behaviour, Device

## Abstract

**Background:**

Wearable activity monitors (WAMs, e.g. Fitbits and research accelerometers) show promise for helping health care professionals (HCPs) measure and intervene on patients’ activity patterns. This study aimed to describe the clinical use of WAMs within South Australia, barriers and enablers, and future opportunities for large-scale clinical use.

**Methods:**

A descriptive qualitative study was undertaken using semi-structured interviews. Participants were HCPs with experience using WAMs in South Australian clinical settings. Commencing with participants identified through the research team’s professional networks, snowball recruitment continued until all identified eligible HCPs had been invited. Semi-structured interviews were used to explore the research aims, with quantitative data analysed descriptively, and qualitative data analysed thematically.

**Results:**

18 participants (physiotherapists *n* = 8, exercise physiologists *n* = 6, medical consultants *n* = 2, and research personnel recommended by medical consultants *n* = 2), represented 12 discrete “hubs” of WAM use in clinical practice, spanning rehabilitation, orthopaedics, geriatrics, intensive care, and various inpatient-, outpatient-, community-based hospital and private-practice settings. Across the 12 hubs, five primarily used Fitbits® (various models), four used research-grade accelerometers (e.g. GENEActiv, ActivPAL and StepWatch accelerometers), one used Whoop Bands® and another used smartphone-based step counters. In three hubs, WAMs were used to observe natural activity levels without intervention, while in nine they were used to increase (i.e. intervene on) activity. Device selection was typically based on ease of availability (e.g. devices borrowed from another department) and cost-economy (e.g. Fitbits® are relatively affordable compared with research-grade devices). Enablers included device characteristics (e.g. accuracy, long battery life, simple metrics such as step count) and patient characteristics (e.g. motivation, rehabilitation population, tech-savvy), whilst barriers included the HCPs’ time to download and interpret the data, multidisciplinary team attitudes and lack of protocols for managing the devices.

**Conclusions:**

At present, the use of WAMs in clinical practice appears to be fragmented and ad hoc, though holds promise for understanding patient outcomes and enhancing therapy. Future work may focus on developing protocols for optimal use, system-level approaches, and generating cost-benefit data to underpin continued health service funding for ongoing/wide-spread WAM use.

**Supplementary Information:**

The online version contains supplementary material available at 10.1186/s12913-021-07096-7.

## Background

Physical inactivity is a major public health problem, with national surveys from the US, UK and Australia indicating that only 33 to 63 % of the adult population meet guidelines for recommended amounts of weekly physical activity [1–3]. Moreover, sedentary behaviour is associated with an increased risk of an array of chronic health conditions, including obesity, type 2 diabetes, cardiovascular disease, some cancers and depression [4]. Clinical guidelines for the management or prevention of chronic health conditions, such as diabetes and cardiovascular disease, include the promotion of regular, moderate-vigorous intensity physical activity as core components [5, 6]. Indeed, the promotion of physical activity as a part of achieving a healthy lifestyle is universally accepted as essential for public health efforts to curb the increasing burden of chronic conditions globally [7].

The routine assessment of patients’ physical activity levels by health professionals is a critical precursor to any intervention. In fact, exercise has been touted as a ‘vital sign’ [8], sparking the emergence of brief, self-report questionnaires for assessing the frequency, intensity and volume of recent physical activity accrued by patients [9]. These brief assessments of physical activity are attractive in clinical practice as they are easily obtained and time-efficient. Yet, self-report is hampered by reporting biases and inadequate reliability and validity compared to objective methods, such as body-worn accelerometers [10, 11].

Wearable accelerometer-based sensors for tracking physical activity levels have traditionally been expensive and designed specifically for research use. However, the relatively recent emergence of cheap, easy to use and consumer-orientated sensors, such as Fitbits, has made accelerometry-based assessment of physical activity available to the general population. The market for wearable activity monitors (WAMs) is proliferating, with the number of wearable units shipped growing by 1275 % from 2014 to 2020 [12], and an estimated $2.8 billion spent on WAMs in 2020 [13]. WAMs offer a wealth of opportunities for health professionals and their patients to objectively assess and intervene on physical activity levels, either as a means to manage pre-existing or prevent chronic health conditions and improve their overall health and well-being.

To date, most research relating to wearables activity monitors has focused on technical and measurement-related issues with WAMs [14, 15], and their effectiveness for intervening on physical activity in various populations, including clinical populations. Recent systematic reviews suggest that WAMs are effective for increasing physical activity in a variety of clinical populations, including patients with cancer [16], type 2 diabetes [17], and those undergoing cardiac rehabilitation [18]. However, relatively little attention has been paid to practical issues impacting the use of WAMs in clinical settings. Factors such as device selection, cost, data management, Health Care Professional (HCP) burden and system-level barriers can all potentially impact the utility of WAMs in clinical practice. Despite the rapid rise of WAMs in observational and interventional clinical research studies [19], and anecdotal reports of their use by health professionals, the extent and nature of their use in current clinical practice is not well understood. To date, a small number of studies have explored the perspectives of patients or health professionals on the potential utility of WAMs for special populations, such as older adults or cancer patients [20–24]. A further study explored health professionals’ perspectives on the use of WAMs following an intervention research trial [25]. To our knowledge, no previous studies have attempted to map the scope and scale of use of WAMs in clinical practice, anywhere in the world. Our study aimed to describe the extent and nature of activities where HCPs are using WAMs with patient groups within the Australian state of South Australia, and understand the experiences, needs, and beliefs of health professionals regarding the use of WAMs in clinical contexts. This included identifying barriers and enablers to their use, and the views of health professionals on the potential future opportunities to integrate WAMs into clinical practice.

## Methods

### Study design and research team

A descriptive qualitative research design was employed involving semi-structured interviews with HCPs. This method captures the candid experiences of participants, and does not generate or explore theories or concepts [26]. Findings of this study are reported according to the Consolidated Criteria for Reporting Qualitative Research (COREQ) [27]. Ethical approval was provided by the University of South Australia’s Human Research Ethics Committee (protocol number 203069). All participants provided informed consent. 

### Participants and recruitment

Participants were HCPs practising in the state of South Australia, with past or current experience using WAMs in clinical practice, or who were preparing to do so in the near future. We included HCPs using both research and consumer-oriented WAMs, and WAMs of any make or model. We excluded HCPs using pedometers or heart rate monitors if they were not used in conjunction with accelerometer-based WAMs; basic pedometers were excluded because we undertook the study to inform the possible future widespread integration of WAMs into clinical practice, which would rely on devices’ “smart” features. Likewise, traditional heart rate monitors (that do not incorporate activity tracking) were excluded since they are designed to solely capture physiological data during exercise sessions, rather than overall activity levels.

 Participants were initially identified through the research team’s professional networks, with snowball recruitment used to identify further eligible HCPs in participants’ networks. As the extent of potential participants eligible and accessible for participation was unknown, we aimed to continue recruitment until data saturation was reached, or no new participants could be identified.

Potential participants were contacted by email inviting them to take part. Those who responded affirmatively completed a telephone or Zoom interview at a mutually convenient time. Where no response to the initial email was received within two weeks, a follow-up email was sent. Where no response to the follow-up email was received within two weeks, contact attempts were discontinued.

### Interview development

A semi-structured interview guide was developed, comprising a combination of open- and close-ended questions (Additional file 1). Interview items captured participant demographics and clinical experience. These were followed by items exploring their experiences and perspectives regarding WAM use in clinical settings, including issues such as patient compliance, the cohesion of WAMs with clinical practice, technical challenges, successes and failures, and other methods used for assessing daily activity levels. At the end of the interview, participants were given the opportunity to add any additional information that they felt was not covered in the questions. The interview guide was piloted with the first three participants, after which the research team met and reviewed the interview transcripts and agreed the interviews were exploring the research topic suitably. These participants had provided consent for their data to be included in the analysis as already described. Thus these participants were retained in the dataset and the interview guide remained unchanged for the subsequent participants.

### Data collection

Interviews were conducted by KS, a research assistant and practicing exercise physiologist, and CM a professor in population and digital health with extensive experience in qualitative research. Data collection occurred between May and September 2020 via Zoom [28] or telephone, depending on participants’ preferences. At the beginning of the interview, the participants were informed that the interview was being audio recorded, and the participant ethics information and consent forms was read aloud to them. To provide informed consent, they were asked to state “I agree”, followed by their name. Brief notes were taken during and immediately post-interview to record interviewee’s impressions. Interviews typically lasted for 20–50 min. Interviews were audio-recorded, and transcribed using the automated Descript transcription software (Descript, San Francisco, CA), which were then checked and corrected by KS. Participants were not reimbursed for participating.

### Data analysis

A manual, iterative, qualitative content analysis approach was employed [29], and data organised using Excel spreadsheets (Microsoft Corporation, Redmond, Washington). This was independently undertaken by each member of the research team initially and involved repeated reading of transcripts and assigning codes and headings to describe the content of interviews. The team met to evaluate other members’ coding and discuss conflicts or different views to reach consensus. This process was repeated to ensure consistent coding across all team members, and then the process was independently conducted by KS. The codes were organised into categories (complete set of categories identified are available in Additional file 2), and then grouped into themes (devices in use, reasons for use, and barriers and enablers to effective use, and future possibilities) which addressed the research questions. Emergent categories and themes were then reviewed by the team members to determine any conflicts with differing views that were resolved through discussion to reach a consensus. Exemplar quotes were extracted to support themes. Data regarding participants’ profession, setting and patient population, device/s used, and metrics of interest were analysed descriptively.

## Results

### Participants

Data collection continued until all known members of the eligible population had been contacted to participate in the study. Thirty-four potential participants were contacted, of whom, 18 agreed to participate. Eight were ineligible (*n* = 3 were primarily involved in research and did not have clinical experience with WAMs; *n* = 3 declined because they did not have any experience using WAMs e.g. they had no experience with activity monitoring technology, or used other technology such as heart rate monitors, but not WAMs; *n* = 2 were senior HCPs overseeing a service which used WAMs but did not have direct experience with WAMs (and so they referred us to other members of their team who had relevant experience). Two participants declined, stating that their close colleague had already participated and they felt they had no additional information to contribute. Six potential participants did not respond to repeated attempts to contact them. To our knowledge, four of the potential participants who did not respond were not direct colleagues of the included participants. Participants’ professional role and setting, and WAMs in use are provided in Table 1.

**Table 1:** Participants’ professional role and setting, and WAMs in use

Participant number	Profession	Setting/context: patient population	Device	Metrics of interest
1	Physiotherapist	Hospital (inpatient - day and home rehab): orthopaedic; stroke; traumatic injuries; post-surgical functional decline Research (community): orthopaedic; stroke; traumatic injuries; post-surgical functional decline	Fitbit Zip® Patients’ own if available StepWatch™ (research)	Steps
2	Physiotherapist, researcher	Hospital (inpatient - day and home rehab): Orthopaedic, neurological, post-surgical functional decline, cancer	Fitbit Zip®	Steps
3	Physiotherapist (Director Physiotherapy and Exercise Physiology)	Hospital (inpatient - day and home rehab): Musculoskeletal/orthopaedic; aged and palliative care; post-surgical functional decline	Fitbit Zip®	Steps
4	Physiotherapist	Hospital (inpatient - rehab): Orthopaedic; stroke; post-surgical functional decline; functional decline medical	Fitbit Zip® Patients’ own if available	Steps
5	Physiotherapist	Hospital (inpatient - home rehab): Orthopaedic; stroke; functional decline medical; cardiac rehab	Fitbit Zip®	Steps
6	Physiotherapist	Hospital (inpatient - rehab): Brain injuries	Fitbit Zip® Patients’ own if available	Steps
7	Physiotherapist	Hospital (inpatient - rehab): Stroke	Fitbit Charge®	Steps
8	Exercise Physiologist	Community clinic: Stress management; ‘overtrainers’	Whoop Band and app	HR; HRV; Activity intensity minutes; Energy expenditure; Sleep/recovery
9	Exercise Physiologist	Community clinic: Musculoskeletal injuries; metabolic	Whoop Band and app Clients’ own if available	HR; HRV; Activity intensity minutes; Energy expenditure; Sleep/recovery
10	Exercise Physiologist	Community clinic: Workplace injuries (musculoskeletal injuries and trauma, amputations, fractures); neurological conditions; brain injuries; intellectual disabilities;	Patients’ own if available Purchase through insurer - typically Fitbits or Garmin	Steps Activity intensity minutes Sleep
11	Exercise Physiologist	Community clinic, Hospital (inpatient rehab): Arthritis; diabetes; musculoskeletal injuries; cardiovascular; post-traumatic stress disorder	Fitbit (couldn’t remember model Patients’ own if available	Steps HR Activity intensity minutes
12	Exercise Physiologist	Community clinic: Obesity (pre-bariatric surgery); metabolic; cardiovascular Hospital (inpatient rehab): Stroke	Fitbit Charge® Patients’ own devices	Steps Activity minutes
13	Exercise Physiologist	Hospital (inpatient - home rehab): Stroke	Fitbit Zip® Patients’ own if available	Steps Activity intensity minutes
14	Researcher, Medical doctor (Anaesthetist)	Hospital (ICU): ICU survivors; significant cardiorespiratory and cardiovascular conditions	Smartphone app (iPhones) Fitbit One®, Fitbit Flex®	Steps; GPS data
15	Researcher, Physiotherapist	Hospital (ICU): ICU survivors	ActivPAL™ GENEActiv	ActivPAL™: Activity minutes GENEActiv: Steps
16	Researcher	Hospital (orthopaedics): Orthopaedic	GENEActiv	Activity intensity minutes; Energy expenditure
17	Researcher	Hospital (orthopaedics): Orthopaedic	GENEActiv	Activity intensity minutes Energy expenditure
18	Medical Doctor (consultant geriatrician)	Hospital and hospital-at-home: Geriatric	ActivPAL™	Activity minutes; Sleep Gait speed

*HR* heart rate, *HRV* heart rate variability, *GPS* global positioning system, *ICU* intensive care unit

Of the 18 participants, eight were physiotherapists, six were exercise physiologists, two were specialist medical consultants, and two were hospital-based research associates. Together, the 18 participants represented 12 discrete hubs of WAM usage (herein referred to as hubs). The hubs included a hospital-based rehabilitation service (which provided inpatient, day-patient and home-based services); a private exercise physiology practice; an orthopaedic inpatient service; two inpatient rehabilitation services (from which one participant had additional experience with WAMs in a community-based exercise physiology clinic); two community-based exercise physiology clinics; two intensive care units (ICU); and a geriatrics service (hospital and hospital-at-home settings).

### Devices in use

Across the 12 WAM hubs, a variety of devices were reported, with some hubs reporting they used more than one type of device. Five hubs reported using Fitbits (Fitbit Inc., San Francisco, CA, USA) (Fitbit Zip® in two hubs, Fitbit Charge® in one, both Fitbit One® and Fitbit Flex® in one, and an “unknown” Fitbit model in another). Four hubs reported using research-grade accelerometers: GeneActiv (ActivInsights Ltd, Kimbolton, Cambridgeshire, United Kingdom) in two hubs, ActivPAL™ (PAL Technologies Ltd., Glasgow, UK) in two hubs, and StepWatch™ (Orthocare Innovations, Mountlake Terrace, WA, USA) in a single hub. Three hubs primarily used patients’ own devices and four further hubs used patients’ own devices in addition to other available devices, which were mostly various models of Fitbits and Garmins (Garmin Ltd, Olathe, KS). One hub used the Whoop band (Whoop Inc, Boston, MA), and the final hub used patients’ smartphones’ inbuilt step counter in addition to using the Fitbit One® and Fitbit Flex®.

Participants were asked how they had selected the model of WAM in use. Thirteen participants reported that it was simply the model that was available, for example, because they were left over from a previous research study, they were provided by the company/business, or because they were able to borrow that particular device from research collaborators (e.g. from a university). Eight participants – some of whom had knowledge of how devices were selected and some of whom were involved with device selection – reported using Fitbits because they were cheap and simple to use. The two hubs using Fitbit Zips reported that the ability to attach the device to different body parts (such as shoes or waist) was a reason for using that device.

*“So having something around the wrist, doesn’t pick up steps as much when you’re using a frame. And so that’s why we liked the [Fitbit] Zips, because if they were on the shoe, they could actually still pick up the step, even if it was a really slow step, and if you have no arm movement and if it was on the wrist or even sometimes on the hip, it wasn’t picking up as much as the shoe.”* (Hospital exercise physiologist)

Another participant reported selecting the WAM was chosen because interstate colleagues used that model in a similar clinical initiative. No participants described a comprehensive process to select the WAM in use.

### Metrics of interest

While WAMs can collect a wide variety of metrics, by far, the most commonly reported metric of interest was steps, reported by thirteen participants. Step count was reported as the sole metric of interest by eight participants (across three hubs) and the primary metric of interest for five other participants (across seven hubs). The second most widely used metric was physical activity minutes (reported by nine participants), which was typically used in conjunction with other metrics, and predominantly amongst exercise physiologists. Small numbers of participants reported using various other metrics: sleep (*n* = 4), energy expenditure (*n* = 4), heart rate (*n* = 3), GPS (*n* = 1) and cadence (*n* = 1).

Typically, the research-grade WAMs were used to capture observational activity data, which was downloaded retrospectively. In contrast, Fitbit, Whoop bands and other patient-owned devices were used for real-time feedback, and used as a clinical tool during therapy encounters, e.g. for goal-setting with patients.

### Reasons for use

Of the 12 WAM hubs, six hubs (mostly community-based exercise physiology clinics) used WAMs purely as a clinical tool, without research motivations. Five hubs initially began using WAMs as part of a clinical research project. Of these, two reported that the WAM use had continued after the end of the project, while three reported that WAM usage had declined or completely stopped since project completion.

In most cases, participants reported the WAMs were used to facilitate goal-setting, inform exercise prescription and promote self-management.

*“So I have had the experience in this role where I’ve had patients who have actually had a device already, and their goal has been made very much centred around that device”* (Community exercise physiologist)

*“Exercise prescription is a highly complex activity undertaken by both exercise physiologists and physiotherapists around matching patient capabilities and then going forward, monitoring the outcome of the initial prescription and trying to match the capacity that they can continue to improve in. You know, I think the primary purpose of our wearables is to actually get that right. And I think it’s something that a lot of physios and exercise physiologists aren’t potentially getting right at the moment.”* (Physiotherapy hospital director)

*“There’s two components to it. One is how do we manipulate what we do so that we’re hitting the training session closer to the ideal mark, but much more importantly is how we coach the client for them to self-manage their health behaviours over the week when they’re not with us.”* (Community exercise physiologist)

Only three hubs reported using WAMs for objective monitoring of activity levels without an intervention component, though one of these hubs was planning an intervention component for future use. All but one participant described WAMs’ ability to collect objective data as a key reason for using them, avoiding the biases associated with self-reported activity measurement approaches.

*“And there are a lot of people … who can’t self-report on that sort of stuff. Or where we’re really not sure - have we got anywhere in the last two weeks with them being on their feet more? So that’s where we really hope that the clinicians could get more idea”* (Hospital physiotherapist)

*“Another benefit of using the devices for assessment is that they provide an objective measure [in contrast to] things like questionnaires and subjective information that is a bit more subject to bias.”* (Hospital physiotherapist)

*“I think recently a medical student was on the geriatric rehab ward, just observing every 30 minutes [and recording] if the patient’s walking, if the patient’s sitting out of bed, is the patient in bed. But to me that’s quite crude data. . – [that’s only] half a second of observation in that 30 minute window. How applicable is it? So I think there is definitely a gap from just visual [observations].”* (Gerontologist)

The ability to use WAMs to monitor patients’ activity levels and progress remotely (e.g. in-home rehabilitation and community settings) or outside therapy sessions was also commonly cited.

### Barriers and enablers

Participants reported a number of barriers and enablers, at the device, patient, staff (therapist and multidisciplinary team), and system levels. Device characteristics such as perceived accuracy, being easily attached to different body sites (e.g. on the wrist or shoe), ability to wear for extended period, long battery life, and having easy-to-use software and simple output metrics were reported as enablers.

*“… if we want to get accurate steps then … it needs to be worn on the … ankle or the foot … I mean there’s little to no movement of the arm at all when they’re using a frame.”* (Hospital exercise physiologist)

*“I can’t actually remember how long - but it does certainly hold [its charge]… at least, or longer than 10 days… We don’t want the clients or patients [to] actually do anything extra. So it just stays on, then it can be set and forget.”* (Gerontologist)

*“I think the more [metrics] it shows…or those that have more functions, becomes harder [for patients to understand]. I don’t know how many quite understand the difference between … active minutes and steps.”* (Community exercise physiologist)

Conversely, devices perceived as being inaccurate (e.g. not detecting steps in slow ambulators), requiring regular charging, or that clipped on to clothing (making them susceptible to falling off or being misplaced with clothing changes) were problematic.

*“That it had to be clicked onto the shoe. [that way it worked] with patients who walked down to 0.4 or 0.5 metres per second, [they] could wear it on the shoe and it would still record a step. But [for] patients that were walking really, really slowly, then it wouldn’t work at all.”* (Hospital physiotherapist)

*“I keep forgetting to charge it. So the charge is probably a big one.”* (Community exercise physiologist)

[About shoe-worn devices] *“But I think one of the detriments to that is we lost a few in the toilet, with trouser pants.”* (Hospital physiotherapist)

Three hubs that provided Fitbits to their patients (as opposed to patients owning their own) described barriers related to syncing/downloading data. For example, HCPs reported having to log in and out of multiple devices linked to a single account as being complicated and time-consuming. In some instances (e.g. community-based services), patients were taught to use the Fitbit, and expected to sync it to their personal phone, but difficulty completing syncing was reported as a barrier.

WAMs were reported as being particularly useful for certain populations/types of patients. For example, they worked best with patients who were: confident with technology, had previous experience with WAMs and were motivated with their therapy.

*“We had a number of Fitbits and we taught the person how to download the app onto their phone or iPad [so they could] look at their steps and generate the charts and all the things that you can do with Fitbit. The … younger tech savvy people were quite interested in that and got right into it. Whereas other people, they were just happy enough to read on the Fitbit that they’ve done so many steps in a day.”* (Hospital physiotherapist)

*“It sort of depends on the individual and how – some people are, sort of get a bit obsessed by their steps and everything. And those are quite good patients to do it with.”* (Hospital physiotherapist)

Many participants reported that providing patients with clear instructions on how to use and manage devices and having procedures in place to manage practicalities were important enablers to circumvent practical obstacles, such as difficulties with charging, syncing, or wearing the WAM incorrectly.

*“And so they, the phone, they were set up to synced accounts for me, so they didn’t need to sync it and say “how do I send this one to my thing?“ I just say “don’t worry about it. You don’t need to. All you need to do is wear this device on a daily basis and charge it whenever it needs to be charged”. And we’d give them a printout schedule saying that over the next week, this is the night that you need to charge the device on, and here’s the charging cable. And here’s a USB plug for it to plug in to.”* (Intensivist)

Specific patient groups were reported as being less well suited or unsuitable for WAMs. For example, several participants indicated against using WAMs with patients with impaired cognition (e.g. with dementia or head injuries), leading to difficulty managing WAMs (e.g. forgetting to put it on, or limited ability to understand the outputs and engage with goal-setting).

*“We have so many people with cognitive deficits … who are so overwhelmed or not able to even deal with normal therapy, let alone adding on something else to the session.”* (Hospital exercise physiologist)

Participants reported avoiding the use of WAMs altogether in certain patient groups due to safety concerns: patients with eating disorders where WAM use may compound dysfunctional exercise behaviour, and patients who needed assistance to ambulate where WAM might encourage unsafe independent ambulation.

*“Some of our patients who are obsessive, who have had eating disorders in the past, might be anorexia and have now on the other end of the eating disorder spectrum. Who, if we’re too prescriptive without intervention, it can actually be detrimental. So we’re not encouraging them to weigh all the time and we’re not encouraging to weigh and measure food. And so the exercise prescription, if it’s a, if they’re an obsessive exerciser, if they take on that obsessive sort of exercise component, then I’d probably say no.”* (Community exercise physiologist)

*“They’ve got to be walking. Ideally the ones that are walking independently. Yeah. Or if they’re sort of what we call standby with the frame, then we could do that as well. We just have to be wary that if we put this on, we want an increase (in) your walking. If they’re standby, we don’t want them to be, you know, walk by themselves without supervision. Cause that’s defeats the purpose, if they might fall over. We’ve got to pick our patients.”* (Hospital physiotherapist)

Many participants described staff-related factors as important barriers and facilitators. The enthusiasm and motivation of the treating HCP regarding the use of WAMs was considered an important facilitator for their effective use, and a barrier to use when this was lacking.

*“I also think it’s on the advocacy of the therapist as well, whoever’s talking to them. Cause I think that there’s, if the clinican is, promoting that, and is enthusiastic. You know, can demonstrate some benefit from wearing one then it’s, yeah. You know, it would be more likely to, for the patient to then uptake that as well.”* (Hospital physiotherapist)

Competing demands of HCPs were reported as a barrier to effective use. Many participants acknowledged that sometimes WAMs took a lower priority than some other clinical duties, particularly when time was limited.

*“Just the work involved people struggled with in terms of, okay, make sure this data is uploaded and then make sure that we’ve got this display ready for, to look at with the client and that sort of stuff. But the little bits that have to fit into the day, are just more things on top of the other thousand things to fit into the day. So that as a, you know, sometimes it’s easy for people to do a short burst of activity, but to try and build it in as a routine thing to keep going, that obviously it was, you know, it didn’t work. It was hard to do and it ended up petering out.”* (Hospital physiotherapist)

The wider therapy and multidisciplinary teams’ attitudes and capabilities were also regarded as important. For example, having nursing staff, the medical team, and auxiliary staff (e.g. cleaners) aware of the WAMs was vital to maximise data completeness and effective use (e.g. reminding patients to wear WAMs consistently and reinforcing activity goals), and avoid device loss.

*“And you’re relying also on other therapists, so whoever’s in the gym, to check that is it on there, is it on there or nurses. That sort of thing or just to make sure they’ve got it on. Which patients are supposed to have the Fitbit on”* (Hospital physiotherapist)

Some participants reported having encountered HCPs and other staff members who were resistant to the use of WAMs, due to scepticism about their value or concerns about the WAMs duplicating (and potentially replacing) in-person therapy.

*“I think what I’m getting at there, I suppose is some of the tools that come in, when we start to adapt them, can provide a threat. I think, to health professionals, and they think, you know, if the patient has the capacity to do this, why do they need me? Sort of thing, which, which I think is not a real threat. And therefore I think the uptake can be relatively poor in some of those, some professional thinking, because they think, well, I’m doing that anyway… So I think that’s part of a threat that potentially limits uptake.”* (Physiotherapy hospital director)

The rate of device loss varied widely between participant accounts. Whilst most reported it wasn’t an issue, two participants reported high rates of device loss (70 % and 40 %, respectively); in one instance, this was attributed to significant difficulty for patients to manage devices due to cognitive impairments and lack of support from other health care staff. In another hub, patients were intentionally discharged home with the WAM and were supposed to return it at outpatient follow-up appointments; however, high device loss rates were experienced. Participants in other hubs reported that device loss was uncommon. Participants in these hubs typically reported having procedures in place to track WAMs (e.g. a booking system) with clearly designated roles and responsibilities assigned to team members (e.g. a particular team member being responsible for collecting the WAM the day before discharge).

*“We have a technology sort of admin person who sort of monitors where all the, our technology stuff is like iPads and other bits of technology. So they sort of tend to remind us what’s happening with this, and who’s got that. And if they have to go out and collect things from people’s homes, they will. They don’t really like to, but I think someone’s sort of onto that already”* (Hospital physiotherapist)

### Future directions

The vast majority of participants were optimistic about the ongoing utility of WAMs in clinical settings. In particular, they viewed that WAMs could make therapy sessions and goals more objective and measurable, and could extend therapy offerings beyond traditional face-to-face delivery (e.g. WAMs could be used to support telerehabilitation or longer-term follow up, to allow to provide additional therapy at lower cost than face-to-face services).

*“The hospital costs will be reduced, but the, the allied health will be remote. And, I think that’s where wearables will come in as well.”* (Hospital-based research associate)

Some participants also commented on the value of WAMs in the large-scale measurement of patient activity patterns. Such datasets may support the future development of risk stratification and prognosis prediction in patients.

*“The next step is really interesting me now as well, actually. This data is going to be incredibly powerful, predicting deterioration, risk stratifying patients, better informing patients about what their outcomes will look like in a way that’s meaningful to them and to the clinician who’s treating them.”* (Intensivist)

Whilst participants were generally positive about the value of WAMs in clinical settings, they commonly cited funding barriers (for example, difficulty obtaining funding for new services and approaches).

*“I think, if you’re trying to sell wearables - why wearable should be used more - you’re going to have to sell “well, it’s going to take [cost] out of this area.”* (Hospital-based research associate)

Participants recommended that more evidence demonstrating the benefits and cost-effectiveness of WAMs will be needed to convince hospital administrators that WAMs are worthwhile.

*It’s a challenge to sustainability because, like everyone else, the health service has limited budget and if you’re just continually buying the same thing over and over, questions do get asked. But you know, it’s, ‘what’s the cost benefit analysis?’ I haven’t done it… But I think that the benefit is probably greater than the cost. If people are using them effectively and getting the input from our professional group that is required to make the use maximised from that effectiveness perspective.”* (Physiotherapy hospital director)

Some participants even commented that the paradigm underpinning the current healthcare model is problematic, and that a shift is needed with greater emphasis on physical activity and lifestyle-changes for illness prevention, and that within such a paradigm, WAMs offer clear value.

*“That’s the whole problem with our health services. We wait until people are sick, then we intervene. Whether it’s elective surgery or rehab or aged care, or pal[palliative] care, you arrive here because you are unwell. So we’re not a health service, we’re an illness service. And you know, the state government some years ago pulled a lot of money out of the community sector, which was supposedly aimed at primary care… But the impact of doing that is now, we have sicker people arriving all the time because there’s no prevention in the community. So that’s a big philosophical point I’m making there.”* (Physiotherapy hospital director)

Participants also reported the need for well-developed systems and protocols to maximise patient compliance with WAMs and streamline the burden on HCPs associated with administering WAMS (e.g. a system to track WAMs and user-friendly software to assist convenient data download, interpretation and goal setting).

*“I also think it’s challenging to fit in, taking a personal approach, without having some sort of management or systems support as well, to be able to make things happen, because if it’s up to individual [HCPs], it gets really, really tricky. So I don’t know what technology is available, but anything that could be automated or kind of, you know, supported in a systems way would probably be helpful for all of those things too.”* (Hospital physiotherapist)

Some participants believed that new WAMs, designed especially for the clinical setting, and determining clinically meaningful outcomes are needed to achieve widespread use and integration into ongoing clinical services. Ideally, such devices would be sensitive enough to measure steps in slow ambulators accurately and simultaneously measure other clinically-relevant metrics (such as oxygen saturation). Others believed that existing, consumer devices could be used effectively with appropriate skill and clinical judgement. Further evidence regarding how to most effectively use WAMs for clinical goal-setting, including optimal progression of such programs, and determining meaningful outcomes and thresholds will be beneficial.

*“So I think there’s a clinical ease of use side of it, but there’s probably also a [need to be] clearer on what the… thresholds and what the values are - how much activity did you have to do to prevent a complication or what information is useful to health providers and patients about activity or sedentary behaviour in a hospital setting that would actually then change their behaviour or flag that this patient is at risk.”* (Hospital physiotherapist)

## Discussion

Wearable activity monitors offer promise to increase physical activity and improve health outcomes, yet their uptake and use by health professionals in clinical settings is poorly understood. Our study shows that although WAMs are being used by HCPs, their use is fragmented and driven by individuals or small teams in isolation. In most cases, HCPs are using WAMs as a measurement and feedback tool to increase patients’ activity level. Less commonly, HCPs are using WAMs as a measurement-only tool (e.g. to determine the effect of a surgical procedure on activity levels) without using the WAM as an intervention tool. Participants identified WAMs as offering a range of key benefits, including their utility to objectively measure activity levels, facilitate patient progression, and remote monitoring. Conversely, the cost of devices, lack of time, and difficulty accessing the data from WAMs were identified as key issues limiting their uptake and ongoing use.

Findings showed enthusiasm for the use of WAMs in clinical settings. This is important for two reasons. First, recent systematic reviews indicate that physical inactivity is highly prevalent when people are admitted to hospital, spending 87–100 % of their day sedentary [30, 31]. Secondly, a recent RCT indicated that exercise interventions could improve physical and mental capacity in elderly, hospitalised patients [32]. Given emerging evidence for the effectiveness of WAMs increasing physical activity in various clinical populations [16–18], it appears that WAMs could have a role in the promotion or measurement of exercise in hospital settings.

Our study has identified that HCPs are using WAMs either as individuals or in small teams, driven by local ‘champions’ rather than taking place at a broader, system level. Many of the barriers identified appeared to stem from the ad hoc nature of WAM use, including a lack of understanding from wider team members and lack of structured protocol for loaning, charging and returning devices, as well as how to access and download the data efficiently. This has implications for the continued use of WAMs and patient engagement, which has found that screen format and autonomous communication are important factors influencing individuals’ choices of wearables [33]. For HCPs, some of the issues relating to usability are underpinned by a lack of awareness of what devices are ‘fit for purpose’, and the desire to access low cost off-the-shelf devices for the individual without considering if they can be used to collect data on a larger scale. One example is HCPs creating individual accounts for patients through the WAMs proprietary software; a time consuming and inefficient way to log and access data in an already time-poor clinical environment. Importantly, there are system level solutions to some of these barriers including staff training, development of protocols and software to aggregate and display patient data. Indeed, the aggregation of data from WAMs for clinical research applications is already occurring (i.e. Fitabase, Verisense), providing opportunities for wider-scale use in the future. Some consumer-level devices also offer application programming interfaces (APIs), providing opportunities for third parties to develop programs to use data from WAMs for custom applications.

In keeping with the fragmented nature of WAM use, a range of devices were being used ranging from consumer-level (e.g. Fitbit) to research-grade (e.g. ActivPAL). The most popular activity metrics, however, were extremely similar despite differences in devices. Most HCPs indicated interest in step count and physical activity related metrics such as minutes spent performing physical activity and activity intensity. There was, however, an apparent dichotomy between a large amount of information some HCPs wanted versus many metrics being seen as too complex or time-consuming to interpret. This parallels WAM users’ perspective in a previous study, which also suggested that step count was the most popular metric [34]. Furthermore, there was considerable interest from HCPs in capturing a broader range of physiological information than many WAMs provide, such as blood pressure, heart and respiration rate. In the past ten years, there has been a rapid rise in the development of wearable patient monitoring technologies for the diagnosis, monitoring and prediction of a range of health-related events [35]. Indeed, consumer-level devices are already providing these data (i.e. ECG monitor in Apple Watch Series 4), though the validity of such data is often unclear. This suggests that WAMs will likely, in future, form part of an overall patient monitoring ecosystem, either as standalone devices or as ‘multi-sensor’ devices. However, given the difficulties many participants conveyed regarding accessing and interpreting data from WAMs, how this information is presented and used in HCP-patient encounters will require further development.

There are several strengths to this study. To our knowledge, this study is the first to describe the use of WAMs in clinical settings and identify barriers and enablers to their use. In-depth interviews were conducted, and we contacted the entire known population of eligible participants in South Australia. This yielded a wealth of information on a challenging, but important, topic.

There are also limitations to this study that require acknowledgement. Six potential participants couldn’t be reached. It is unclear whether they would have contributed additional insights or if they would have truly been eligible - in our experience, fewer recommended participants met the inclusion criteria as recruitment progressed and the pool of potential new participants diminished. Finally, due to limited resources, we did not do the data analysis in duplicate, however our team worked together closely to identify categories and themes. The mapping exercise was limited in scope to South Australia. However, many of the findings do not appear to be specific to the South Australian context and so would seem likely to be relevant elsewhere.

### Implications

It appears WAMs have a valuable role for optimising therapy and identifying areas of need. The role of WAMs in clinical practice will likely grow as home- and community-based health services become more prominent. However, the full potential of WAMs in clinical practice is not currently being realised due to lack of coordination in their use across the health system.

HCPs who participated in this study were either using research accelerometers or consumer-orientated WAMs, neither designed for day-to-day use in clinical settings. Accordingly, a number of the barriers to use of WAMs identified by our participants are likely to be caused or exacerbated by the fact that the research-grade and consumer-orientated WAMs are being used in a different manner to that which they were primarily designed for (e.g. the time-consuming initialisation and data download processes associated with research accelerometers, and email-linked/mobile phone pairing for consumer-orientated WAMs). Furthermore, the “bottom-up” approach to WAMs in clinical practice (i.e. WAMs being championed by sole or small teams of HCPs, without system-level supports) means there is often a lack of established protocols and multidisciplinary team support to facilitate the optimal uptake and use of WAMs. In future, the potential for system approaches to WAMs in clinical practice should be explored. This may underpin the development of a more standardised approach to the use of WAMs in clinical practice, with the potential for centralised and routine data collection. To achieve this, the following future research is recommended:

1. Scoping of the level of interest in/support for a universal approach to WAMs in clinical practice amongst key stakeholders at multiple levels of the health system (e.g. HCPs, hospital administrators, health department).
2. Identify the key characteristics of a WAM (both the device and its accompanying software) for it to be well suited to use in various clinical contexts.
3. Undertake an audit of existing WAMs and software platforms to determine whether an existing product can meet these needs, or if not, decide which adaption is needed.
4. Work with the multidisciplinary team to develop protocols and training supporting the use of WAMs in a range of clinical settings.
5. Small and large scale trials examining the effectiveness and cost-benefit of WAMs in a range of clinical populations and settings.

Addressing these areas rigorously and in a real-world manner will require close collaboration between researchers and healthcare stakeholders.

## Conclusions

At present, a range of health professionals working across various patient populations and healthcare settings are using WAMs in their clinical practice. However, usage is occurring in discrete “hubs”, with no coordination between hubs in terms of the wearable device used or protocols for use. Many of the barriers to using WAMs in clinical practice may be minimised or removed through more coordinated efforts with system-level supports. In future, a universal approach to WAMs in clinical practice, with centralised data collection, could create vast opportunities for health service and outcome improvements. Ongoing collaboration between researchers and healthcare stakeholders will be needed to realise this potential.

## Supplementary Information

**Additional file 1.**

**Additional file 2.**

## Data Availability

The raw data from this study will not be shared because participants’ did not give their permission for this. Carol Maher should be contacted for requesting the data.

## Abbreviations

APIs: Application programming interfaces
COREQ: Consolidated Criteria for Reporting Qualitative Research
HCPs: Health care professionals
ICU: Intensive care unit
WAMs: Wearables activity monitors
