# Supplementary material for: The use of accelerometer-based wearable activity monitors in clinical settings: current practice, barriers, enablers, and future opportunities
Source: BMC Health Serv Res. 2021 Oct 8;21:1064. doi: 10.1186/s12913-021-07096-7 (PMC8501528; doi:10.1186/s12913-021-07096-7)
Supplement: Supplementary file 1 — Additional file 1. [file 12913_2021_7096_MOESM1_ESM.docx]

**Interview guide**

**Date:**

**Interviewer:**

| **Introduction** | Overview of study and gather informed consent |
| --- | --- |
| **Professional details** | Profession; Years of experience; Work setting (clinic, hospital, aged care etc) |
| **Attempts made** | What attempts have you made to integrate wearable activity monitors into practice?  Probes:  Which device(s) have you used?  Which populations have you used wearable activity monitors on?  What protocol(s) have you used?  How frequently do you use activity monitors? |
| **Reason for use** | Why are you/have you been interested in using wearable activity monitors in clinical practice?  How did you choose the device(s) and protocol(s) you have used?  Probe:  What are the benefits of using wearable activity monitors in health care?  What are the shortcomings of using wearable activity monitors in health care?  Why should clinicians be using wearable activity monitors? |
| **Patient compliance** | What is the follow-up/return rate of monitors?  Are wearable activity monitors easy for patients to use?  Probe:  Have your patients been compliant with the use of wearable activity monitors?  Do you think patients want to use devices?  Do you think devices are burdensome for patients? |
| **Cohesion with current practise/Technicalities** | Is the data obtained from wearable activity monitors cohesive and relevant with the working models and setting that you work in?  Do you think that available wearable activity models are cohesive with the working models and settings that health professionals work in more broadly?  Probes:  Does the data obtained from monitors provide the information you require (e.g. physical activity levels, sedentary time, sleep)?  Is the data obtained from monitors simple to understand or does it require further evaluation and analysis to obtain relevant information?  Are there important data or activity parameters (e.g. intensity of physical activity, exercise modality) that monitors are unable to capture? |
| **Successes and failures** | What are some of the successes and failures you have had when using wearable activity monitors with patients?  Probe:  What has worked?  What hasn’t worked? |
| **Other methods of assessing daily activity patterns** | How else do you assess daily activity patterns in your patients?  Probe:  Do you use questionnaires to assess physical activity/sleep?  Do you use other objective measures do you use to assess daily activity patterns?  What subjective information do you gather relating to daily activity patterns? |
| **Participation in future studies** | Would you be interested in participating in future studies on the use of wearable activity monitors? |
| **Other topics discussed** |  |
| **Post-interview comments or leads:** | Including potential participants recommended |
