# Supplementary material for: The use of accelerometer-based wearable activity monitors in clinical settings: current practice, barriers, enablers, and future opportunities
Source: BMC Health Serv Res. 2021 Oct 8;21:1064. doi: 10.1186/s12913-021-07096-7 (PMC8501528; doi:10.1186/s12913-021-07096-7)
Supplement: Supplementary file 2 — Additional file 2. [file 12913_2021_7096_MOESM2_ESM.docx]

**Themes and Categories**

| **Theme** | **Subtheme** | **Category** |
| --- | --- | --- |
| Devices | Device selection | Word of mouth recommendation |
|  |  | availability |
|  |  | Cost |
|  | Metrics of interest | Steps |
| Reasons for use |  | Goal setting |
|  |  | Exercise prescription |
|  |  | Promote self-management |
|  |  | Objective activity data |
|  |  | Remote monitoring |
| Barriers and enablers | Device factors | Accuracy |
|  |  | Attachment |
|  |  | Extended wear |
|  |  | Battery |
|  |  | Software |
|  |  | Simplicity |
|  | Patient factors | Tech-savvy |
|  |  | Motivation |
|  |  | Impaired cognition |
|  |  | Eating disorders |
|  |  | Requiring assistance for ambulation |
|  | Clinician factors | Clinician motivation and enthusiasm |
|  |  | Competing demands and time constraints |
|  |  | Multi-disciplinary involvement and support |
|  |  | Perceived threat |
|  | System factors | Procedures for managing device use |
|  |  | Need to cost-effectiveness data |
| Future directions |  | Improve clinical practice |
|  |  | Funding and support required for ongoing use |
|  |  | Healthcare paradigm shift |
|  |  | Streamlined systems |
|  |  | Device requirements |
|  |  | Further research |
